# Combined spectral and speech features for pig speech recognition

**DOI:** 10.1371/journal.pone.0276778

**Published:** 2022-12-01

**Authors:** Xuan Wu, Silong Zhou, Mingwei Chen, Yihang Zhao, Yifei Wang, Xianmeng Zhao, Danyang Li, Haibo Pu

**Affiliations:** 1 College of Information Engineering, Sichuan Agricultural University, Ya’an, Sichuan, China; 2 Department of Economics, University of Calgary, Calgary, AB, Canada; Mae Fah Luang University, THAILAND

## Abstract

The sound of the pig is one of its important signs, which can reflect various states such as hunger, pain or emotional state, and directly indicates the growth and health status of the pig. Existing speech recognition methods usually start with spectral features. The use of spectrograms to achieve classification of different speech sounds, while working well, may not be the best approach for solving such tasks with single-dimensional feature input. Based on the above assumptions, in order to more accurately grasp the situation of pigs and take timely measures to ensure the health status of pigs, this paper proposes a pig sound classification method based on the dual role of signal spectrum and speech. Spectrograms can visualize information about the characteristics of the sound under different time periods. The audio data are introduced, and the spectrogram features of the model input as well as the audio time-domain features are complemented with each other and passed into a pre-designed parallel network structure. The network model with the best results and the classifier were selected for combination. An accuracy of 93.39% was achieved on the pig speech classification task, while the AUC also reached 0.99163, demonstrating the superiority of the method. This study contributes to the direction of computer vision and acoustics by recognizing the sound of pigs. In addition, a total of 4,000 pig sound datasets in four categories are established in this paper to provide a research basis for later research scholars.

## Introduction

With the rise of modern intelligent agriculture, how to grasp the growth status of pigs more efficiently and accurately has become a hot issue in the development of farming industry [1].

The environment in which the pigs are grown, the feeding methods and the farm management practices all influence the growth and health and productive life of the pigs and are directly related to the development of the farm [2]. The sound of the pig is one of its important physical information, which is closely related to the growth status and health condition of the pig. Chung Y et al. found that diseases of the respiratory system in pigs under herd conditions are contagious and prone to herd disease [3]. In the case of African swine fever, for example, the most significant disease symptom after contracting the disease is coughing [4], so the vocal characteristics of pigs can directly respond to the respiratory system. Also sound is considered as a basis for judging the stress state of pigs [5], because sound is more accessible biological information characteristically, and collecting sound information can be done without close contact with pigs, reducing the possibility of triggering additional stress reactions in pigs [6]. By analyzing the sounds made by pigs during growth, the growth status of pigs can be derived and necessary measures can be taken to ensure the healthy state of pigs and avoid stressful behaviors, which can help to protect the health of sows or extend their productive life, and can significantly help to improve the efficiency of the whole farm [7].

With the development of computer technology and wireless sensor network technology, sound recognition technology has been widely used in people’s daily life [8]. DNN speech network models have been successfully applied to a variety of speech classification tasks, demonstrating that high accuracy in speech attribute detection and phoneme estimation can be achieved using DNNs [9]. [10] classify different emotions from different languages by constructing an artificial neural network (ANN). The generation of feature sets is performed using the Mel Frequency Cepstrum Coefficient (MFCC) and Short Term Energy (STE). In the ambient sound classification task, [11] used a novel deep convolutional neural network to extract high-level features from the features of the spectrogram through stacked convolutional and pooling layers, achieving excellent results. [12] constructed a Sichuanese speech recognition system by combining Hidden Markov Model (HMM) and Deep Long Term Memory (LSTM) network to compensate the problem that DNN only captures the context of a fixed number of information items. In addition, the use of pre-trained deep neural network to extract sound features requires the network to discuss the sequence of its features, and then use these features as the input of the LSTM model to classify, so that the model can better define the sequence information, and prove that the effectiveness of the fusion model for sound signal detection is shown [13].

Based on these studies, the sound recognition technology of animals has been developed to some extent. Animal vocalization is a form of animal behavior, and vocalizable animals can communicate effectively within a group by calling. Animal sounds can reflect a variety of physiological conditions such as hunger, pain, or emotional state, and are the easiest of the biological characteristics to capture through non-contact means. Therefore the use of sound for the analysis of behavior, health and animal welfare has gradually become an important method [14]. [15] proposed a two-stage audio visual target detection method by using the sound signal of a cow as an RGB image, which achieved better accuracy compared to other methods. [16] introduced an end-to-end feed-forward convolutional neural network that can reliably classify the source and type of macaque calls in a noisy environment using two audio data streams. [17] proposed a Hidden Markov Model (HMM) system for the automatic classification of African elephant vocalizations. Other scholars have used the Mel frequency cepstral coefficient and Gaussian mixture model to identify four different species of individual birds and achieved the desired results [18]. [3] extracted Meier cepstral coefficients (MFCC) from pig sound data and used support vector data description (SVDD) and sparse representation classifier (SRC) as classifiers, respectively, and achieved 91% classification accuracy. [19] combined the commonly used clustering methods and the design of various neural networks, determining a set of center points in the spectrum, processing the features in the spectrum, calculating the similarity space, and using the dissimilarity vector for classification. This combination of supervised and unsupervised approaches works well on the bird and cat call datasets. A large number of scholars have also contributed to the study of sound recognition in pigs [20–22]. Transformer was introduced for the first time in the task of pig sound recognition. It combines the network of attention mechanism and goes a step further on the traditional time series model, which makes the model have parallel ability and combines the feature extraction ability of convolutional neural network. Therefore, its model structure It has excellent global feature perception ability and local feature extraction ability, which is more suitable for pig call classification, and also achieves high accuracy in the evaluation of other animal sounds [23]. In addition, noise is a major challenge in speech recognition applications; ambient noise, reverberation, channel interference, and microphone distortion can all affect the data [24].

Most of the existing speech recognition methods extract features by converting speech information into MFCC features and convolution from the obtained spectrograms, and MFCC features are not stable in the presence of noisy data [25]. However, in the complex environment of pig farms, the collected data often have a lot of noise, which affects the experimental accuracy. On the other hand, for speech only, feature extraction from the waveform graph is also prone to feature loss. The waveform graph is a representation of the audio in the time domain. And the spectrogram is the representation of audio in the frequency domain. Time domain and frequency domain are the two viewing surfaces of analog signals. When the signal is analyzed in time domain, some signals have the same time domain parameters, but at this point it does not mean that the signals are exactly the same. This is because the signal not only varies with time, but also with frequency, phase and other information. At the feature level, the data in two different dimensions complement each other’s features to a certain extent. Therefore, a single model construction is not the optimal solution to solve the speech analysis task, and the combination of spectrum and audio shows some superiority. Based on the above discussion, this paper proposes a new idea for the pig sound recognition task by introducing sound data to complement its features in the process of spectral map classification at the same time, and finding a new method for combining audio features and spectral features by combining sound spectral images with audio signals trained under a fusion network. Improving the single network and optimizing the pig breeding environment to enhance the socioeconomic and animal health breeding benchmarks. In this paper, we collected speech datasets from pigs in various situations and designed a fusion network structure to help improve the accuracy of the pig sound recognition task. This paper is structured as follows, and in the second part, we review the relevant methods. The Methods and materials section describes in detail the algorithms and models used in this paper. The Experimental results section focuses on the experimental part, including the representation of the data set, comparison of the model performance, and analysis of the experimental results. In the final section, the paper is summarized and future directions for the work are proposed.

## Methods and materials

### Ethics statement

The protocol of the animal experiment in this paper was approved by the Animal Welfare Committee of Sichuan Agricultural University, which conforms to the ethical requirements of animal welfare and allows relevant experiments to be carried out. (“Affidavit of approval of animal ethical and welfare”, approval number:2020041).

### Spectral feature extraction method

Mel Frequency Cepstrum Coefficient MFCC feature extraction Mel Frequency Cepstrum Coefficient (MFCC) is widely used in the field of speech recognition and is a feature that is widely used in speech recognition [26]. Studies have shown that the human ear is more sensitive to low frequency signals. The relationship between frequency and human ear perception is linear when the frequency is less than 1 kHz and logarithmic when the frequency is greater than 1 kHz. Mel (Mel) frequency is the method of converting the actual frequency from linear to nonlinear. Besides, there are chroma [27], spectral contrast [28], tonnetz [29] and other methods used for audio feature extraction.

### Audio feature extraction method

The speech classification task can also be achieved by directly extracting features from the sound waveform and transforming the obtained acoustic features into many articulated phonemes. The WAV file of the original audio can then be seen as consisting of individual points of the sound waveform. The key to the whole process lies in how to convert the extracted acoustic features into phonemes, a task that is usually solved using temporal models. However, for pigs, there is no specific phoneme library constructed for model learning. So this technique can be used to extract the acoustic characteristics of the animal, but it cannot help people to understand the sound of the pig.

### Recurrent neural networks

Jeffrey L. Elman proposed the simplest RNN model containing a single self-connected node [30]. Recurrent Neural Networks, or RNN, is a deep learning model that models sequence data, sequence data as input, recursion in the evolutionary direction of the sequence and all nodes are connected in a chain-like recursive neural network. However, RNNs are very difficult to train and have limited applications due to the problems of gradient vanishing and gradient exploding. It is not possible to learn long distance dependencies and RNNs cannot effectively use historical information as the distance increases [31].

### Long short-term memory

**Fig 1:**
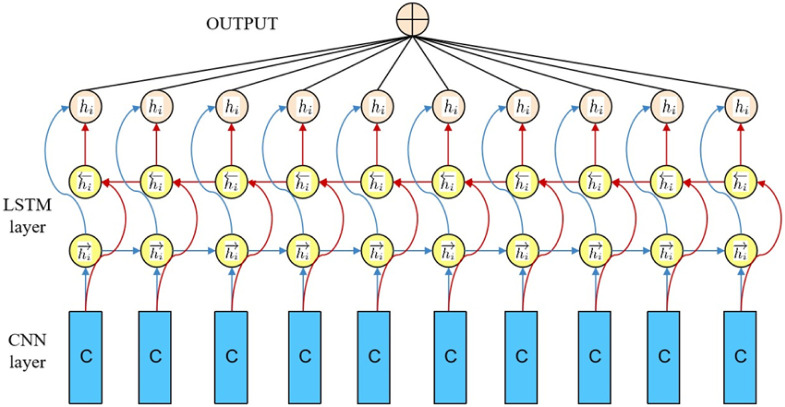
LSTM-CNN structure schematic.

LSTM was proposed by Hochreiter et al. in 1997 and is a special RNN structure [32]. It is able to model the long-time dependencies of the inputs, while solving to some extent the gradient vanishing problem caused by RNNs when back-propagating over longer time series [33]. The module of LSTM contains an input gate, an output gate and a forgetting gate to learn the weights through the collaboration of 3 gates to achieve the effect of being able to store long-term information. CNNs have been widely used in feature engineering due to their ability to note the most obvious features. And LSTM has the property of time-ordered expansion and is widely used in time series. So nowadays, LSTM is often used in combination with CNN, and the structure is shown in Fig 1. For example, Li C et al. proposed a hybrid neural network model combining CNN and LSTM, and introduced an attention mechanism to apply it to stock price prediction, and experiments verified that the proposed model has a better prediction effect [34].

### Gate recurrent unit

GRU was proposed by research scholars in 2014 [34],which is a highly effective variant of LSTM networks, which has a simpler but still effective structure than LSTM networks and can also solve the long dependency problem in RNN networks [35]. Unlike the LSTM, there are only update and reset gates in the GRU model. The update gate is used to control how much of the previous state information is brought into the current state, and the larger the value of the update gate the more state information is brought into the previous state. The reset gate controls how much information from the previous state is written to the current candidate set; the smaller the reset gate, the less information is written to the previous state. The number of parameters of GRU is less than that of LSTM, so the overall training speed of GRU is faster than that of LSTM.

### Voice Activity Detection(VAD) and frame addition window

In sound samples, sometimes there are invalid sound samples, and to eliminate the influence of environmental sounds that appear in the sound samples, it is necessary to use to endpoint detection technique [36]. Endpoint detection is used to determine the start time and end time of the sound, which helps to reduce the training parameters as well as improve the system recognition efficiency. The sound signal is a non-stationary signal, and in the process of processing the sound signal, the processing for the sound is based on the short-time stationary characteristics of the sound signal, and feature extraction is performed for a small segment of the speech signal. Short-time analysis requires the use of frame-splitting and windowing techniques [37]. Treating the sound as smooth at very short times and dividing the audio into equal-length short-time speech frame sequences, for each frame with a window function to multiply, makes the data at both ends of the frame signal get weaker and thus strengthen the data in the center. In order to eliminate the discontinuous waveforms that exist after each frame transformation, the frame signals are panned to overlap each other, compensating for the weakened data at the head and tail ends of the frame.

### Experimental dataset

**Fig 2:**
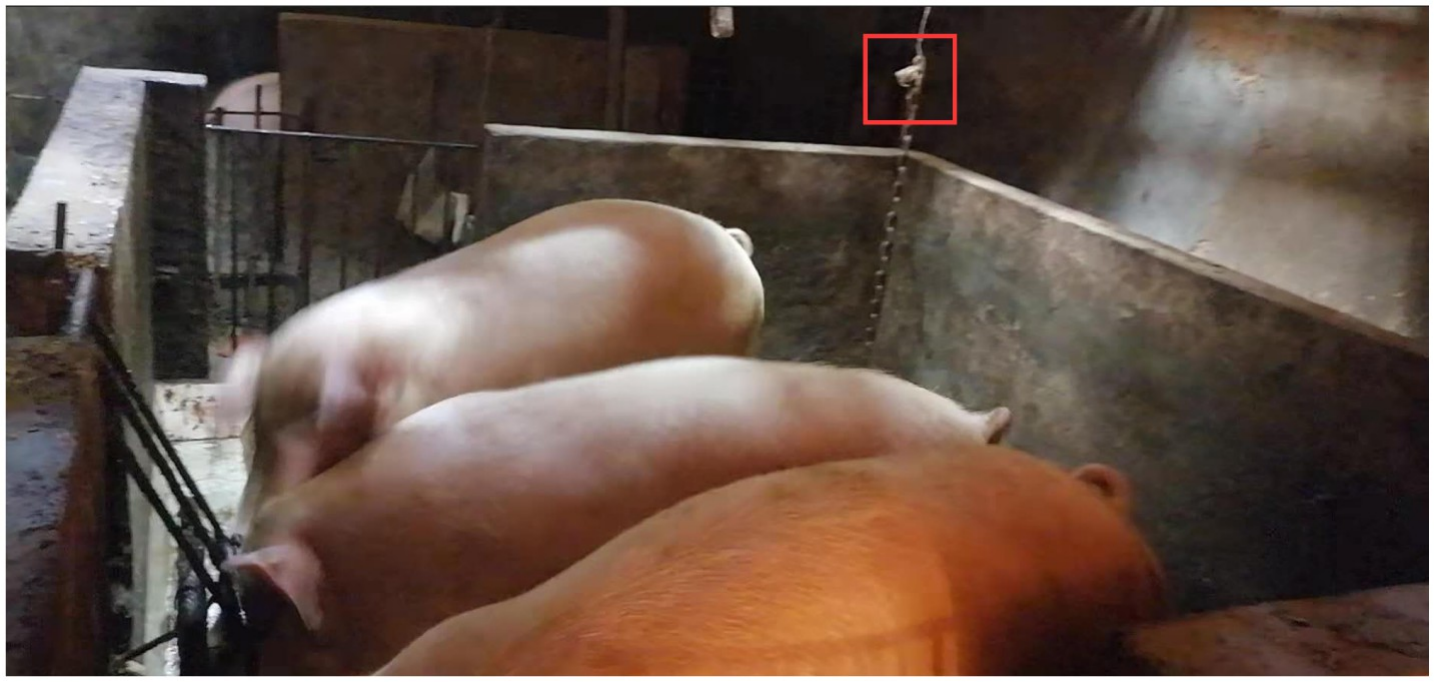
Experimental environment and equipment placement diagram.

The experimental data were collected in a farm in Ya’an, Sichuan Province, China. In the actual environment of a farm, there are situations where multiple pigs are kept in one pen. In this case, multiple pigs will make sounds at the same time and the collected data is often fuzzy with large noise. To ensure the accuracy of the raw data, we identified the experimental target as sows. On farms where the sows are much larger, and where there are often only 1-3 sows in a pen to facilitate childbirth, are the experimental subjects most conducive to obtaining the best experimental results. Also, using sows as experimental subjects to judge their health status and growth condition will have higher economic benefits. The experimental equipment is a recording pen B610 branded as Lenovo, which can make accurate recordings of the sound generated in a 10-meter area. The data obtained from the device is recorded at a bit rate of 512kbps and saved in WAV format for lossless storage. In order to avoid human interference, because it is too close to the pigs leading to stress and other adverse reactions, the recording equipment is placed at a distance of about 1.5 meters from the vertical height of the experimental subject, which will not interfere with the normal life of the pigs. The specific acquisition environment and acquisition equipment are shown in Fig 2.

By consulting with breeding experts, we classified the basic behaviors of domestic pigs, which include the above four categories such as calm, feeding, frightened and anxious. Calmness is obtained for pigs in a normal humming state when there is no stress reaction. The chewing sound made by the pigs during feeding is defined as feeding, and the feeders in the farm are used to feed the pigs intensively at 9 am, 12 noon and 6 pm. Scaring refers to sounds produced by pigs under reinforcing stimuli, such as pigs being driven with sticks or vaccinated. When collecting these sounds, strong artificial stimulation is required, making the collection more difficult and most time-consuming in practice. Anxiety is defined as a grunting sound made by domestic pigs when they are agitated, usually manifested as a stress response to the sight of food, similar to a howling sound. In order to ensure the recording effect as well as to get reliable label sound data, the recording process needs real-time monitoring and preliminary tagging of the recorded audio according to the status of the live pigs to facilitate subsequent processing. In the data classification process, it is ensured that the behavior corresponding to the sound made by the pig is confirmed and labeled by both video and voice at the same time. A total of 4,000 experimental samples were extracted, and the types of sound samples collected were normal grunting, anxiety sound in the state of hunger, feeding eating sound, and howling sound when the pigs were frightened, of which 1,000 experimental data were saved for each sample to support the data set for subsequent experiments as shown in Table 1.

**Table 1:** Database audio volume classification statistics.

Classification	Train set	Test	Total
calm	800	200	1000
feeding	800	200	1000
anxious	800	200	1000
frightened	800	200	1000
Total	3200	800	4000

### Data processing

In the sound data collected, there may be multiple states of sound and invalid sound segments in a segment of audio, and the length of the audio varies, so further manual tagging and batch slicing operations are required to construct the data set required for the experiment. The software used for manual annotation is Audacity audio processing software. Audacity can import and export WAV, MP3, Ogg Vorbis or other sound file formats. It supports the recording and playback of audio files in MP4, MOV and other formats, and can also cut, copy, and paste the sound and undo an unlimited number of times. In addition, Audacity can also perform operations such as envelope editing and noise elimination to meet the data processing needs during the experiment. Based on the above considerations, this paper uses Audacity for audio data processing.

**Fig 3:**
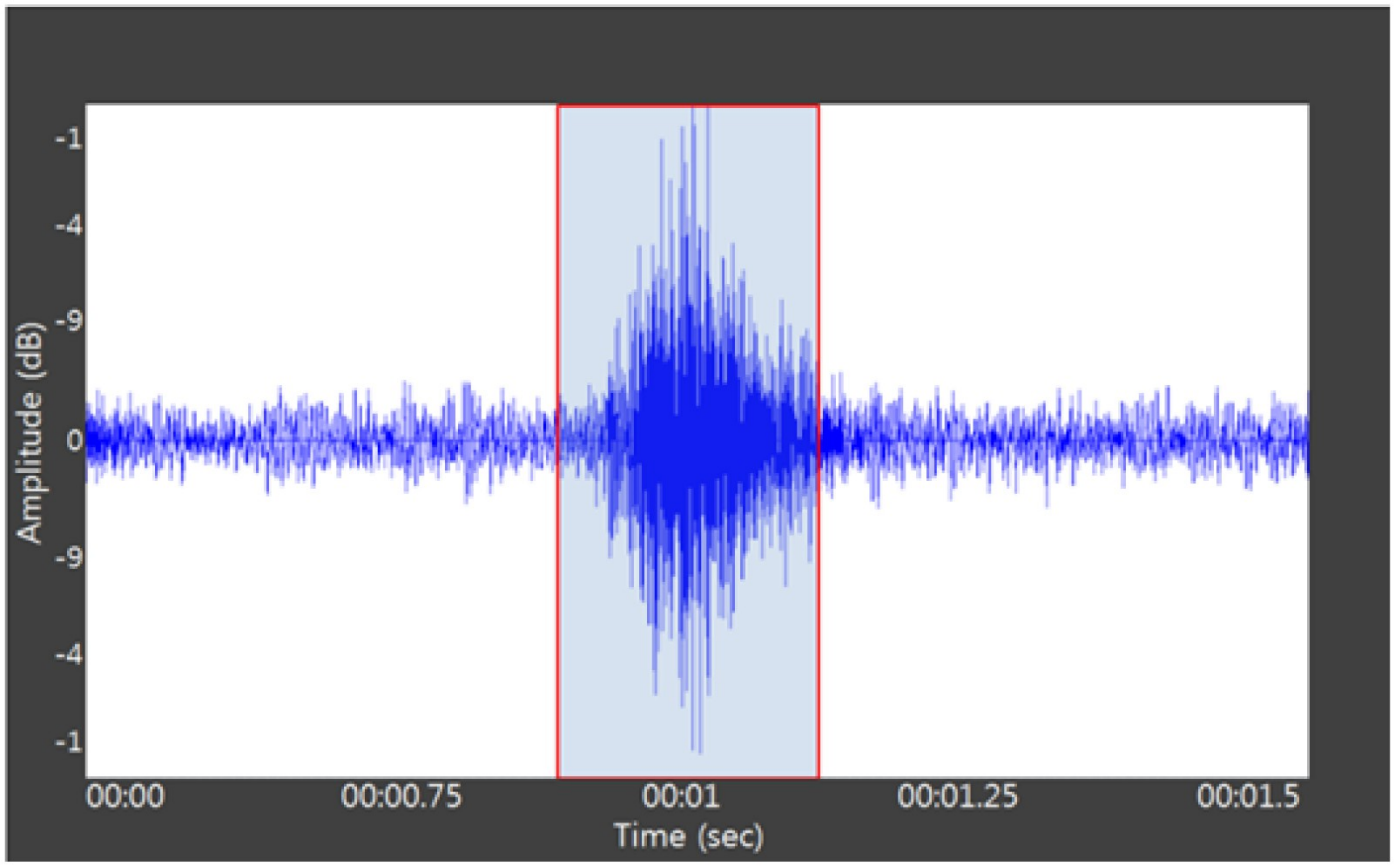
Data processing results graph.

In the process of data labeling, we found that the duration of effective vocalization of each pig was usually between 0.5s-1.8s. We specified the duration of each data as 2 s, and then sliced the whole speech data into one sample with a range of 2s. Since our data volume is large enough, the available data will not be over-fitted to the highly parameterized deep neural network model. In addition, audio data is different from image data, and adding noise data on top of it for data enhancement will have a negative impact instead. The data set processing is shown in Fig 3.

#### Audio data processing

Due to the short-term stationarity of speech signals, we usually frame the speech. For the defined rectangular window function *w*(*m*), then for the speech signal *x*, the speech signal *x*_*n*_(*m*) of the nth frame after windowing and framing is defined as:

$$\begin{array}{c} x_{n}\left(m\right)=w\left(m\right)x\left(n+m\right)0\leq m\leq N-1, \end{array}$$ (1)

The short-term energy En of the speech signal *x*_*n*_(*m*) in the nth frame is defined as:

$$\begin{array}{c} Z_{n}=\sum_{m=0}^{N-1}x^{2}\left(m\right) \end{array}$$ (2)

The short-term zero-crossing rate represents the number of times the waveform signal crosses the zero value in a frame of speech. For continuous signals, zero-crossing means that the waveform passes through the time axis. For discrete signals, zero-crossing means that the sign of adjacent sampling points changes. First define The symbolic function sgn is:

$$\begin{array}{c} sgn\left(X\right)=\{\begin{array}{cc} 1 & ,x>0\\ -1 & ,x<0 \end{array} \end{array}$$ (3)

For the nth frame of speech signal *x*_*n*_(*m*), the short-term zero-crossing rate *Z*_*n*_ is:

$$\begin{array}{c} Z_{n}=\frac{1}{2}\sum_{m=0}^{N-1}\left|sgn\left[X_{n}\left(m\right)\right]-sgn\left[X_{n}\left(m-1\right)\right]\right| \end{array}$$ (4)

Another windowing function is Hamming windows, which is used in this paper as a windowing function for our speech signal processing.

In the part of frame addition window, this paper uses Hamming window as the window addition function, whose weighting coefficient will make the side flaps reach a small range, focusing the energy on the main flap and reducing the side flaps to make them decay slowly. The formula is as follows:

$$\begin{array}{c} w(n)=\{\begin{array}{cc} 0.54-0.48\text{cos}\frac{2\pi n}{L-1} & ,0\leq n\leq L-1\\ 0 & ,else \end{array} \end{array}$$ (5)

where L is the window length and n denotes the number of points of the signal in the window.

#### Spectrum data processing

In the spectral feature processing section, in order to increase the number of features, audio features such as chroma [23], spetral [24] and tonnetz [25] were extracted through the Librosa library, in addition to using log-mel spectrograms and MFCC, the two most widely used auditory features in sound. The features with the best results are selected in subsequent experiments and input to the audio network section to complement the spectral features. The Mel Frequency Cepstrum Coefficient MFCC feature extraction conversion formula is as follows:

$$\begin{array}{c} M\left(f\right)=1125\times \text{ln}\left(1+\frac{f}{700}\right) \end{array}$$ (6)

**Fig 4:**
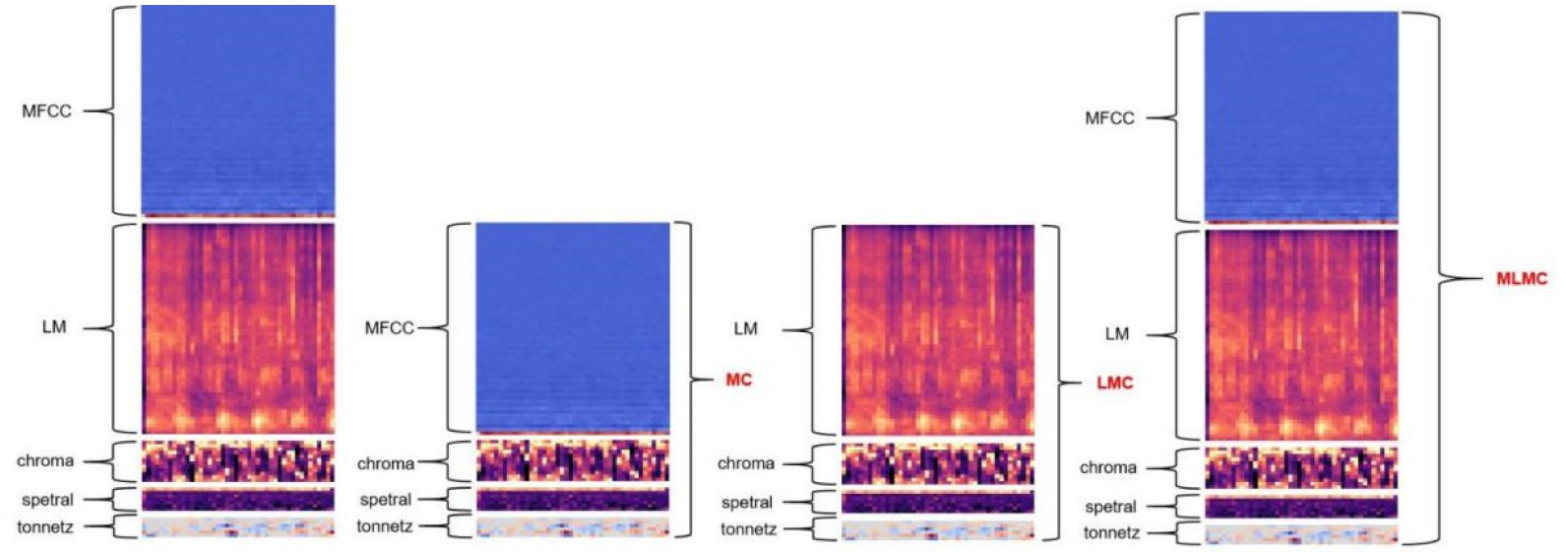
Feature map. (a) Five categories of essential feature. (b) Combined features obtained based on basic features.

The formula M(f) is the frequency and f is the linear frequency. log-mel that is calculated for M(f) taking log. As shown in Fig 3, after obtaining five features such as MFCC features on the raw data, then using Log-mel spectrogram, chroma, spectral contrast and tonnetz combined together to form the LMC feature set, MFCC is combined with chroma, spectral contrast and tonnetz to form the MC feature set. A total of eight candidate features were generated to increase the feature diversity, and the best training features were selected based on the experimental results in order to increase the experimental accuracy, and the feature effects are shown in Fig 4.

### Improved fusion network

**Fig 5:**
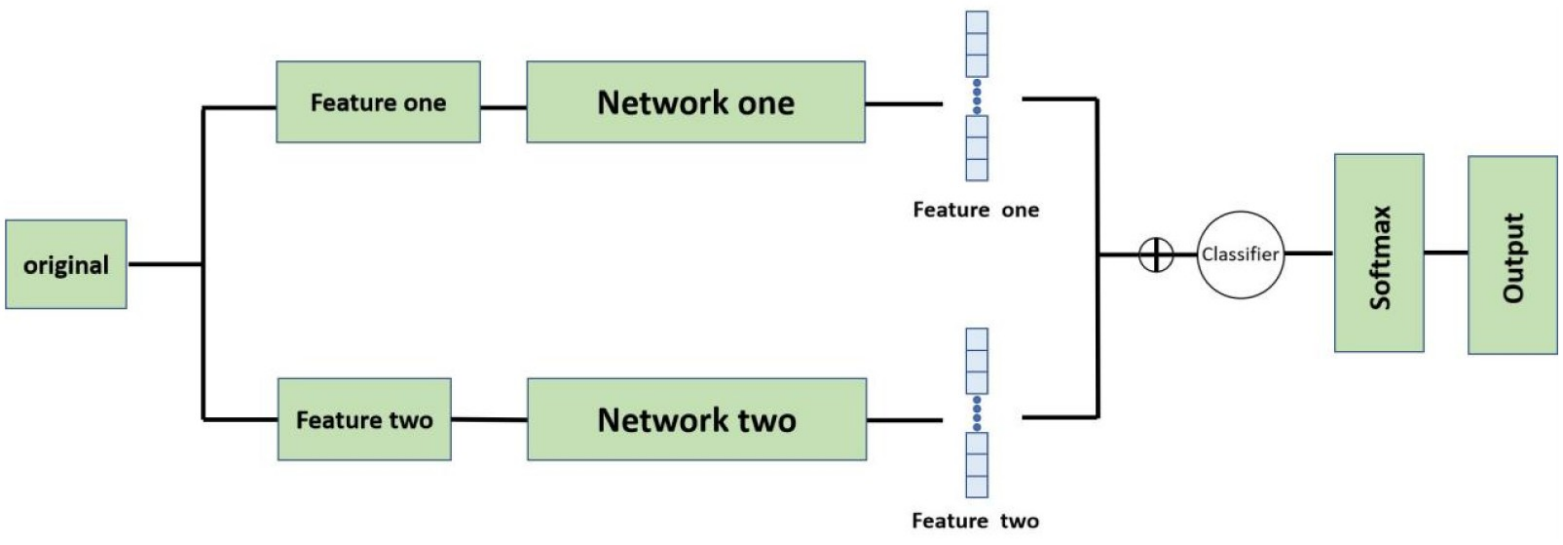
Improved fusion network architecture graph.

In order to make full use of both spectral and audio features, a parallel network structure is designed to train both types of data and fuse the two types of high-level features into the classifier. The upper layer of the network selects the image classification network to perform convolutional feature extraction on the spectrogram data and outputs the convolutionally extracted high-dimensional features. Considering that sound features are temporal in nature, a sequential network is used in the lower layer of the network for training to better retain sound information to ensure the accuracy of the experiment. The two models with the best results are selected for combination during the subsequent experiments, and two primary learners with different input features are trained from the initial dataset, and the high-dimensional features of the two layers are combined to generate a new dataset for training the second learner after reaching the best. The network outputs of the first two layers are treated as sample input features, while the initial sample tokens are still treated as sample tokens, i.e., the combined high-dimensional features from the first two layers of the network are input to the classifier in the latter layer of the network, so the choice of classifier is also exceptionally important. The specific model for each layer of the network is determined after the experiment, and Fig 5 shows the overall network framework.

## Experimental results

In order to verify the effectiveness of the model in this paper, we first spectral features were experimented with audio features, and the prediction results were selected for comparison. ACC is used as the evaluation index, and ACC indicates the accuracy rate, which reflects the number of correctly predicted categories as a percentage of the total number.

$$\begin{array}{c} ACC=\frac{TP+TN}{TP+TN+FP+FN} \end{array}$$ (7)

Where TP represents the number of positive case predictions judged to be correct, TN represents the number of negative case predictions judged to be correct, FP represents the number of negative case predictions judged to be wrong, and FN represents the number of positive case predictions judged to be wrong. In the fusion network section, MSE and MAE are also added as evaluation metrics.MAE and MSE represent the absolute mean error and mean squared error corresponding to each category during the training phase. They are calculated as follows:

$$\begin{array}{c} MAX\left(y,\overset{ˇ}{y}\right)=\frac{1}{n}\left(\sum_{n}^{i=1}\left|y-\overset{ˇ}{y}\right|\right) \end{array}$$ (8)

$$\begin{array}{c} MSE\left(y,\overset{ˇ}{y}\right)=\frac{1}{n}\left(\sum_{n}^{i=1}|y-\overset{ˇ}{y}{|}^{2}\right) \end{array}$$ (9)

where y denotes the true value, $\overset{ˇ}{y}$ denotes the predicted value, and N denotes the total number of samples in total. To judge the superiority of the final trained classifiers it is necessary to add ROC curves for a comprehensive evaluation of each classifier, which works by giving a model input to a set of data with known positive and negative classes and measuring the performance of this model by comparing the predictions made by the model for that set of data. AUC represents the area under the ROC curve, which is calculated as follows:

$$\begin{array}{c} AUC=\frac{\sum_{ins_{i}}\in positiveclass^{rank_{ins_{i}}}-\frac{M\times (M+1)}{2}}{M\times N} \end{array}$$ (10)

where M is the number of positive samples and N is the number of negative samples, where the negative sample book = total number of samples—number of positive samples.

The composite evaluation index F1 is the summed average of accuracy and recall. recall reflects the proportion of positive cases whose data were correctly determined to the total number of positive cases and is calculated as:

$$\begin{array}{c} Recall=\frac{TP}{TP+FN} \end{array}$$ (11)

Precision indicates the proportion of the sample sample classified as positive cases that are actually positive cases and is calculated as:

$$\begin{array}{c} Precision=\frac{TP}{TP+FP} \end{array}$$ (12)

F1 is defined as the summed average of the precision and recall rates and is calculated as:

$$\begin{array}{c} F1-score=\frac{2*Recall*Precision}{Recall+Precision} \end{array}$$ (13)

### Spectral feature experiment

**Fig 6:**
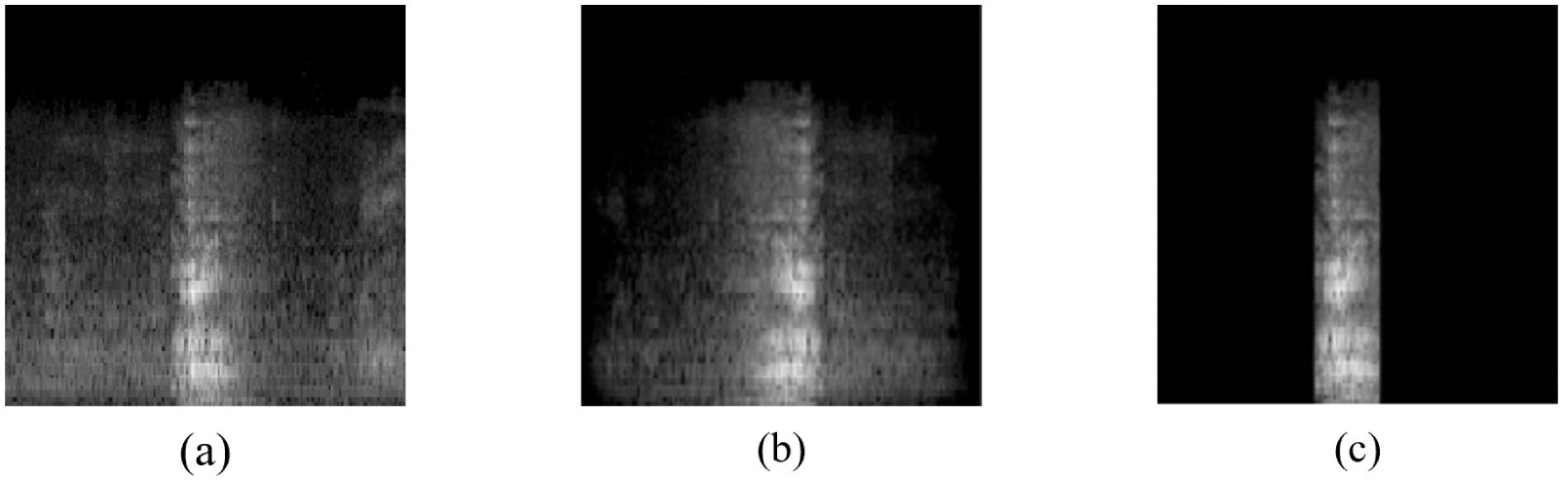
The original data and the processed data are shown, (a) the original data, (b) the windowed sound spectrum, and (c) the endpoint detection sound spectrum.

Seven image recognition models such as Desnet,Resnet50,Resnet152,Effcienet B1,Effcienet B2,VGG16,VGG19 were used to compare the results. Before selecting the best features, a data processing method comparison experiment was conducted in order to compare the accuracy of different data processing methods. The data used in this part of the experiment are all MFCC features, which are the original spectrogram data, the framed and windowed data, and the endpoint detection data, and the input of the defined model is a two-dimensional 224X224 single-channel spectrogram, as shown in Fig 6.

The experimental results are shown in Table 2. The prediction results of the data processed with windowing under the determined model outperform the original data and the endpoint detection processed data.

**Table 2:** Database audio volume classification statistics.

Model	Total params	Origion Data	Add Window	Voice Detection
Desnet	7,041,604	0.7195	0.7184	0.6548
Resnet 50	23,595,908	0.8115	0.8478	0.7359
Resnet 152	58,379,140	0.8045	0.8103	0.6425
EfficientNet B1	6,580,356	0.8116	**0.8563**	**0.8013**
EfficientNet B2	7,774,198	**0.8149**	0.8333	0.7986
Vgg 16	134,276,932	0.7306	0.8173	0.6915
Vgg 19	139,586,628	0.7956	0.8236	0.7316

**Fig 7:**
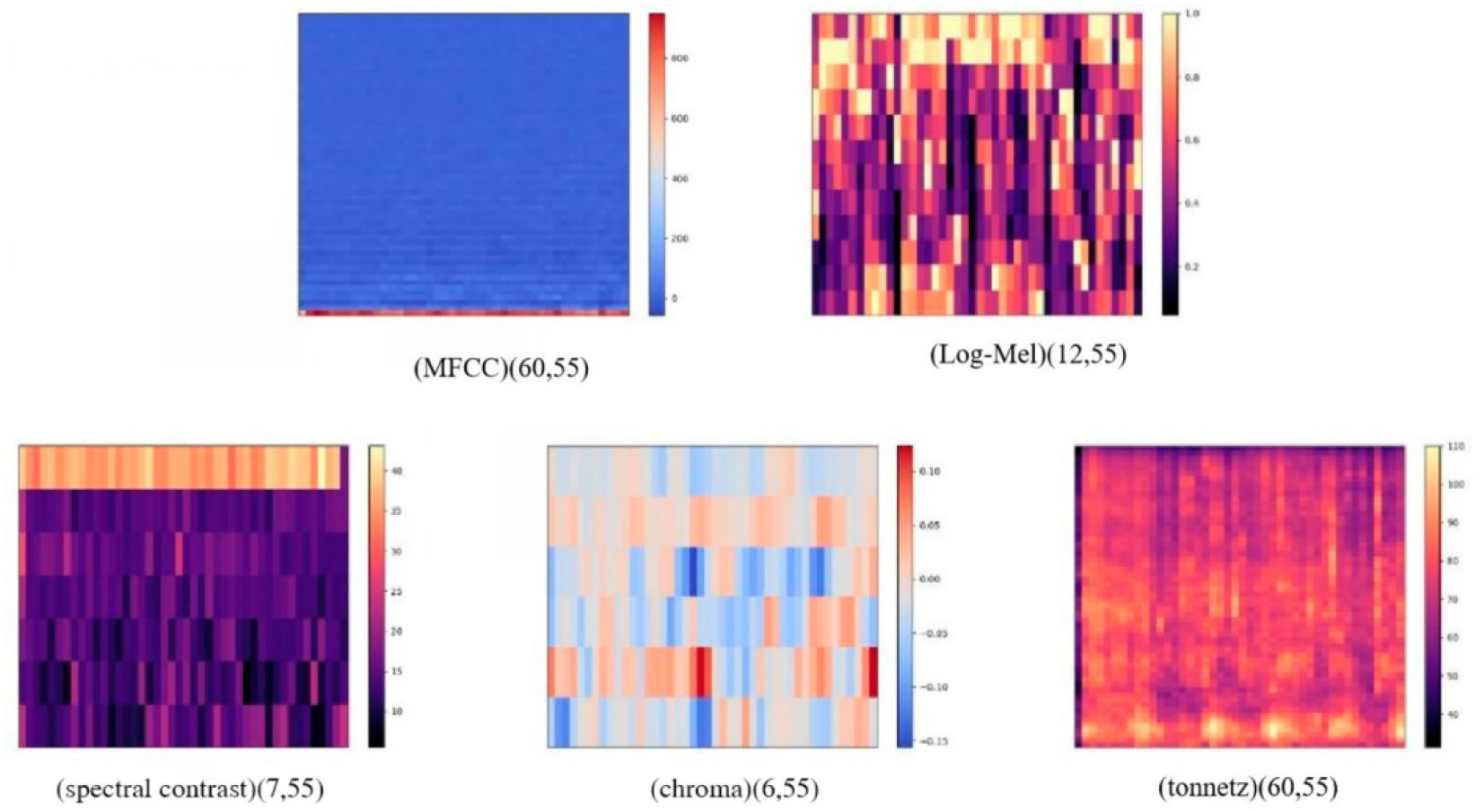
Schematic diagram of the features of the five types of samples.

As the original extracted data contained 4000 sound samples, each sound was 2 s in duration. the dataset recorded four classifications, eating, normal, frightened, and anxious. In the data information extraction stage, we extract the data information into five categories of basic data information, and we define the sampling frequency of the data as 48000. Each sound sample is divided into fixed-length frames, and these frames are overlapped, and a total of five types of features, MFCC, log-mel, contrast, chroma, and tonnetz, are extracted for a specific dimension through the librosa library carried by python, which are the basic features extracted. The features are shown in Fig 7.

After determining the best data processing method again, we discarded several networks with significantly poor results and conducted experiments to select the best features. We expanded the data on the original 5 categories of base features. The five types of basic features are overlapped to generate three types of fused data: MC at 85x85 scale, LMC at 85x55 scale, and MLMC at 145x55 scale. In this part of the experiments, we will test the training performance of 8 classes of candidate features in different neural networks, so as to select the most effective features. The output classes of the model are defined as 4 classes, and the experimental results are shown in Table 3.

**Table 3:** Comparison table of experimental results of different features in different networks.

	Input dimensions	Test accuracy				
		Vgg 16	Resnet 50	Vgg 19	Desnet	EffcienNet B1
F1	(60, 55)	0.6704	0.7500	0.7727	0.8068	**0.8989**
F2	(12, 55)	0.5284	0.6466	0.4545	0.6704	**0.6931**
F3	(7, 55)	0.6079	**0.7672**	0.5681	0.4147	0.7509
F4	(6, 55)	0.6022	0.5113	0.500	0.6136	**0.6170**
F5	(60,55)	0.7329	0.7727	0.7954	0.8125	**0.9103**
Mc	(85, 55)	0.7500	0.78409	0.7954	0.8522	**0.9080**
Lmc	(85, 55)	0.7102	0.78409	0.7727	0.8409	**0.9156**
Mlmc	(145, 55)	0.7386	0.8011	0.8011	0.8409	**0.9186**

The results show that in all feature experiments, there are large differences among the models, and the accuracy of the models is low except for EffcienNetB1. Combining all the features we found that almost the worst performance was achieved on Vgg 16 for various categories of features indicating the low model matching ability of its model in response to the pig speech recognition task cannot correctly respond to the classification task. While Resnet 50,Vgg 19,Desnet are relatively good, the Vgg 19 model only achieves an accuracy of 0.4545 under the F2 feature, indicating that the generalization performance of its model needs to be enhanced, and the other models are also in the corresponding situation. Experimental results based on EffcienNetB1 show that the model can better incorporate audio features, extend the input multiple dimensional data for feature extraction, and utilize more useful information at the same time. For the EffcienNetB1 model, although the accuracy is slightly lower than that of the Resnet 50 model by about 0.01 in the case of the F3 feature, excellent performance is achieved for the remaining seven features, which illustrates the power and applicability of EffcienNetB1. In the course of the experiment we tested the model through four categories and we got the preliminary experimental results. For the eight types of feature data, the small dimensionality of the three types of features, F2, F3, and F4, is not conducive to feature extraction with and subsequent lower dimensional inputs, resulting in unsatisfactory experimental accuracy obtained for the three types of feature data. Based on the above experimental results, this study decided to discard the F2, F3, and F4 data in the subsequent experiments and put more experimental resources into selecting the best experimental results. The results of the two-part test have shown that Efficienet B2 has an 85.63% optimal prediction effect under the frame-splitting and windowing process. In the case of Lmc feature extraction, the whole model achieves 92.86% optimal results. The small number of parameters also saves a lot of time for the experimental process. Compared with the other five networks EfficientNet converges faster at the beginning and EfficientNetB1 has less fluctuation compared to EfficientNetB2. The other networks are very smooth in the training process but converge very slowly and cannot extract feature information effectively resulting in poor final results. The experiments in this section identify EffcienNetB1 as the spectrogram feature classification network, and the default choice of adding windows to the data is made for subsequent experiments.

### Validate the validity of MLMC

In this experiment, the optimal feature MLMC obtained in Table 3 is used as the final evaluation feature. The output MLMC features are passed through the ACC evaluation parameters obtained by EffcienNetB1.

**Fig 8:**
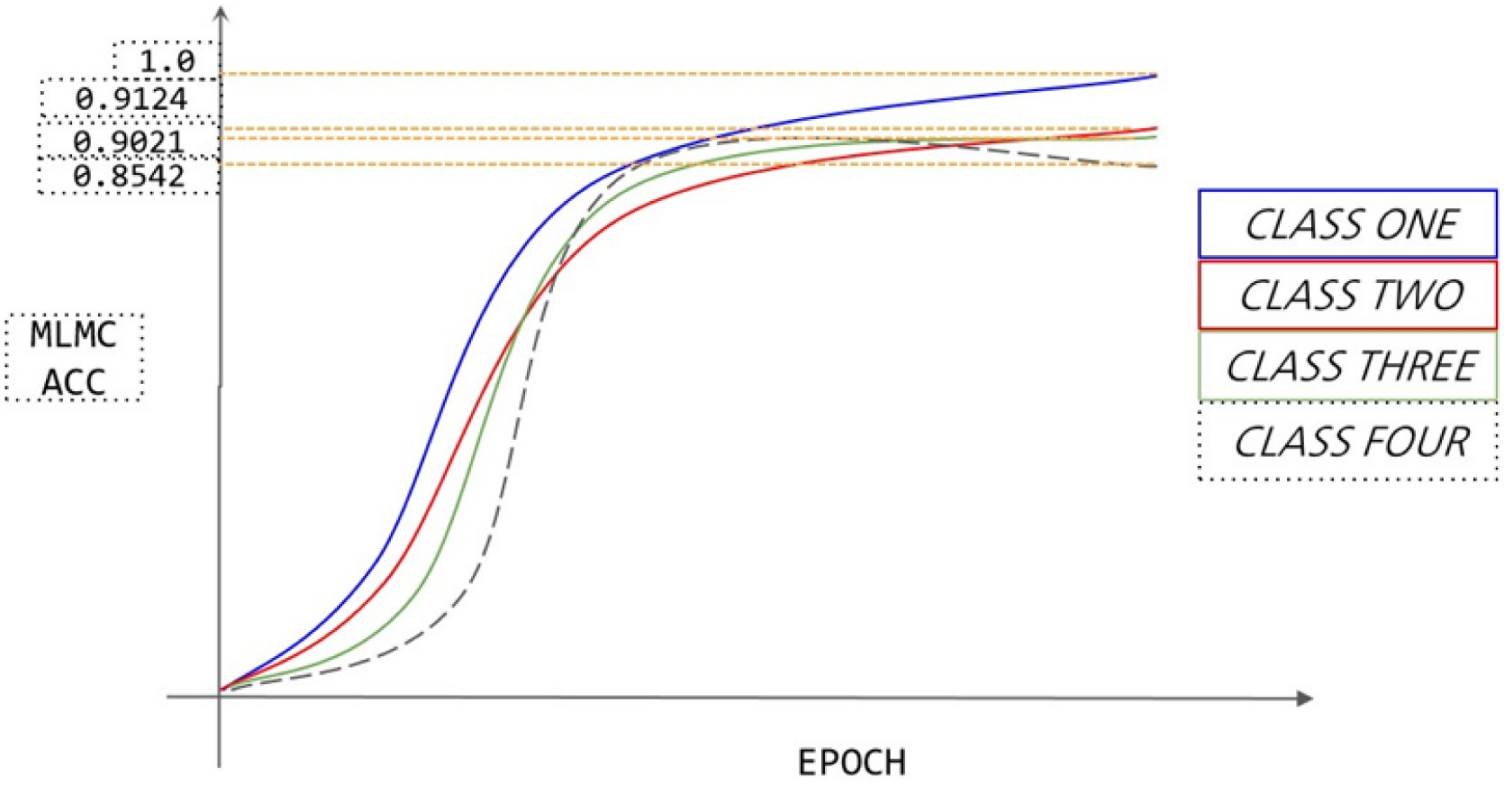
MLMC features in four types of data.

As shown in Fig 8, the MLMC data were evaluated using the EffcienNetB1 model, and output probabilities of 1.0, 0.9124, 0.9021, and 0.8542 were obtained for each of the four types, for a total combined ACC value of 0.917175. Through the result analysis, it is found that the experimental results are poor for the fourth category of data under MLMC features. The fourth category of experimental data is the impatient howling of pigs, and the sound signal behaves sharply and briefly from the spectrogram, which leads to the fourth category of data being more difficult in the experimental feature extraction process and the training effect is relatively poor. Compared with the fourth type of data, the first type of data is the normal grunting of pigs, which is more gentle in the spectrum and is conducive to the feature extraction during the experiment, so the experimental effect is the best among the four types of data.

### Audio feature experiments

**Fig 9:**
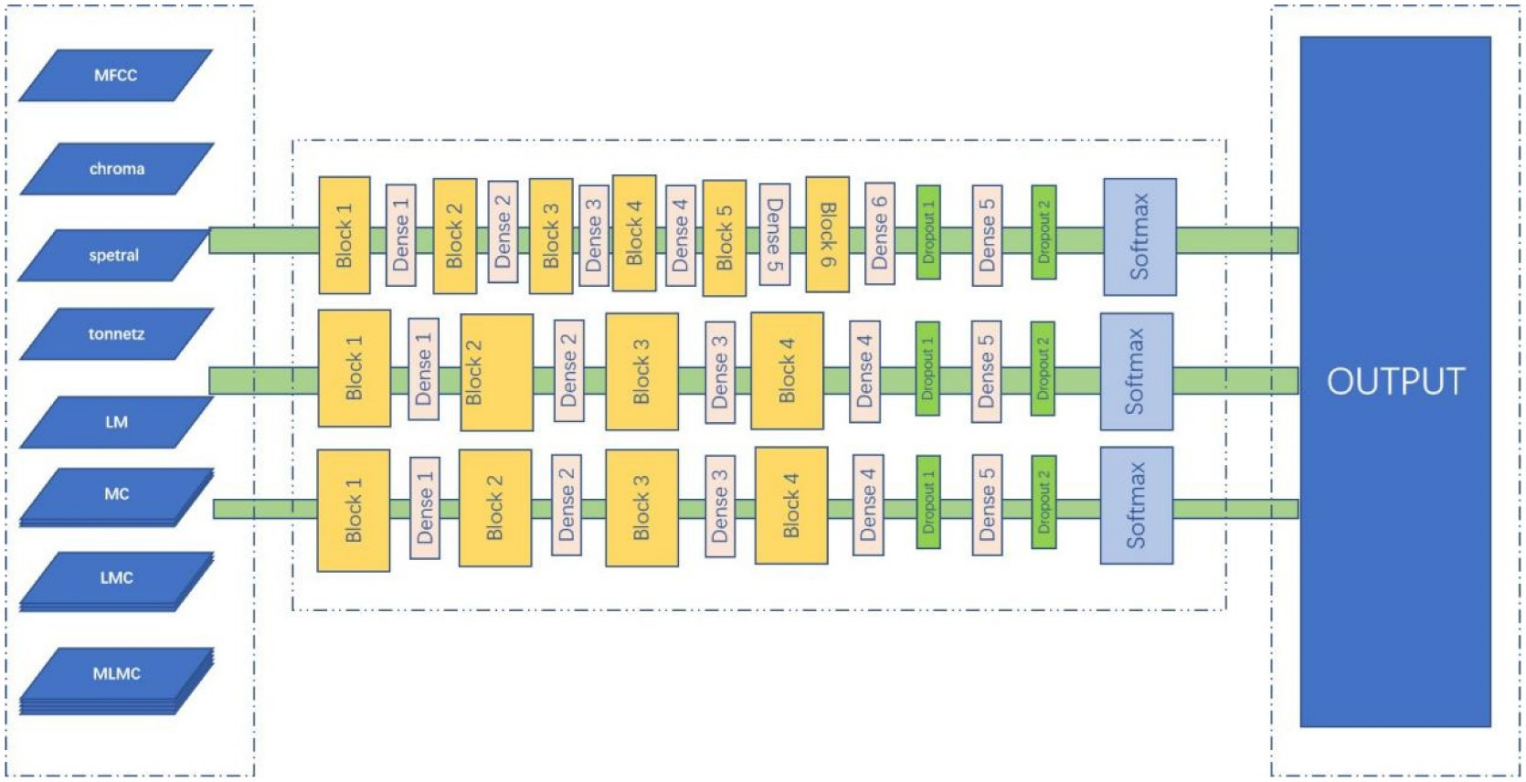
From top to bottom, the network structure of RNN, LSTM, GRU is shown.

In this section, considering the time-series nature of speech data, RNN, LSTM, and GRU networks are selected for comparison experiments, and the network structure is shown in Fig 9.

**Fig 10:**
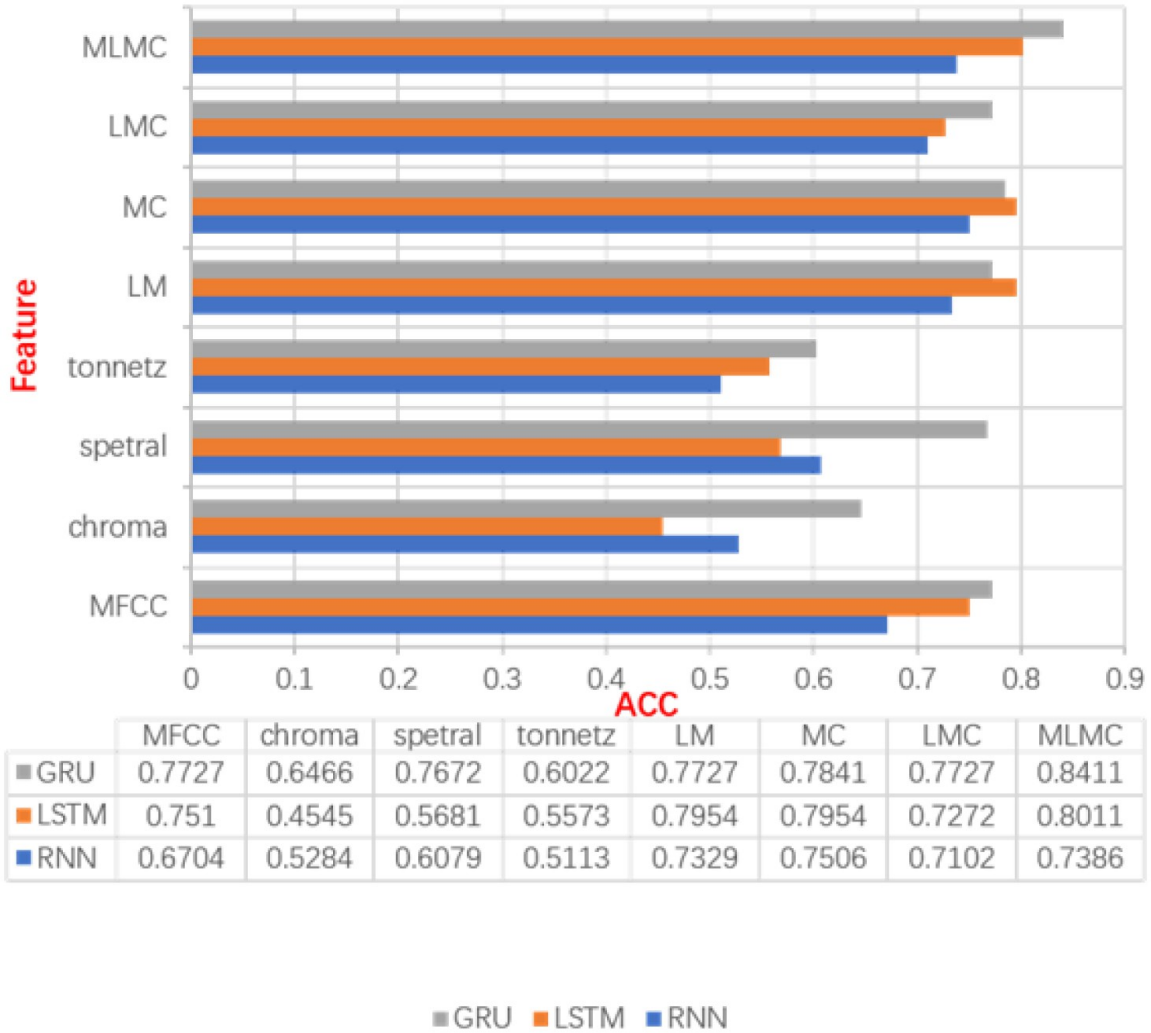
Experimental results corresponding to each type of audio features.

The results, according to Fig 10, show that the highest ACC obtained when selecting MLMC features can reach 0.8411, which is much higher than the extraction results of the other seven features. The MLMC features are a combination of five individual features that contain a variety of audio information from different extraction methods. The large amount of speech feature information helps the network to over solve the classification task better. According to Fig 10, the accuracy of GRU is higher than that of LSTM and RNN under most features in the three sequential networks, but LSTM outperforms GRU in LM and MC features. To further discuss the effectiveness of the three sequential networks, the experimental results are discussed in conjunction with Efficientnet at the same time.

### Improved fusion network experiments

In the discussion of previous experiments, we identified Efficientnet as the classification network for the spectral features and used the addition of windows in the data processing. Since the superiority of MLMC features was demonstrated during the audio spectrum feature selection experiments, the MLMC features with input dimension (145,55) were selected as the default audio features in the subsequent experiments. However, the relevant experiments cannot fully illustrate the superiority of the GRU network. In this section, the experiments put the previous three types of sequence networks into the fusion network separately, and the classifier of the final layer is chosen among logistic regression and SVM, and the network with the best effect is selected according to the experimental results.

According to Table 4, the prediction results of the fusion network are much better than those of the individual spectrogram classification and speech classification networks, which also show greater advantages under MAE, MSE and AUC metrics. Although the training accuracy of the individual networks is high but the testing accuracy is very low indicating that overfitting occurs during the training process, while in the fusion network there is almost no overfitting further proving the superiority of the fusion network. After identifying EfficientnetB1 as the spectrogram classification network, the best test accuracy was achieved when the sequence network was selected GRU classification network selected Logic, but it was slightly lower than the fusion network when LSTM was selected as the sequence model in terms of MAE,MSE metrics. When AUC is used as the discriminant, EfficientnetB1+GRU+SVM outperforms the other networks by virtue of 0.99163, which proves that the classifier of this network works best. Since GRU reduces the number of parameters based on LSTM so choosing GRU as the sequence network will save the experiment time. With the different classifiers, we noticed a significant change in the metrics and had to refocus our attention on LR and SVM. The SVM approach is to learn the classifiers by considering only the support vectors, i.e., the few points most relevant to the classification. In contrast, logistic regression reduces the weight of points far from the classification plane by non-linear mapping, and relatively increases the weight of data points that are most relevant to the classification. In essence, the purpose of both is the same, but after SVM is transformed into a pairwise problem, the classification only needs to calculate the distance to a few support vectors, which is an obvious advantage in the calculation of complex kernel functions, and can greatly simplify the model and calculation, In addition, SVM is better at solving this classification problem.

**Table 4:** Table of experimental results of the fusion network.

Model combination	Test ACC	Train ACC	MAE	MSE	AUC
EffcientnetB1	0.9186	0.9265	0.10834	0.13684	0.92280
RNN	0.6718	0.7641	0.15816	0.19897	0.89951
LSTM	0.7562	0.8925	0.13775	0.18877	0.90147
GRU	0.7758	0.8605	0.17857	0.26020	0.92106
EffcientnetB1+RNN+SVM	0.9207	0.9347	0.03698	0.03698	0.98024
EffcientnetB1+LSTM+SVM	0.9228	0.9335	**0.03443**	**0.03443**	0.98993
EffcientnetB1+GRU+SVM	**0.9339**	**0.9452**	0.03862	0.03826	**0.99163**
EffcientnetB1+RNN+LR	0.9106	0.9133	0.06632	0.07653	0.93284
EffcientnetB1+LSTM+LR	0.9117	0.9184	0.08673	0.10714	0.95217
EffcientnetB1+GRU+LR	0.9136	0.9231	0.09183	0.12244	0.97128

**Fig 11:**
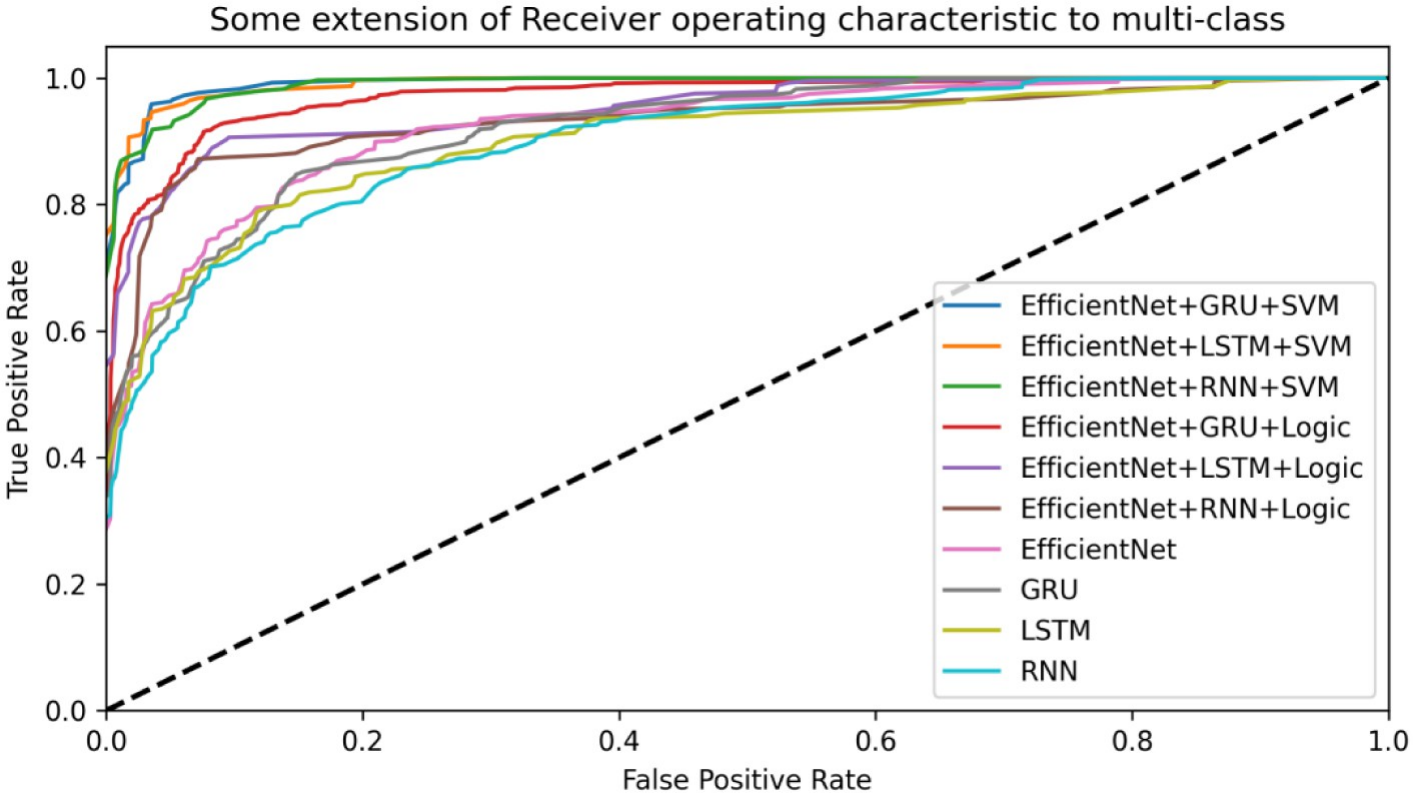
Comparison of ROC curves of each model.

**Fig 12:**
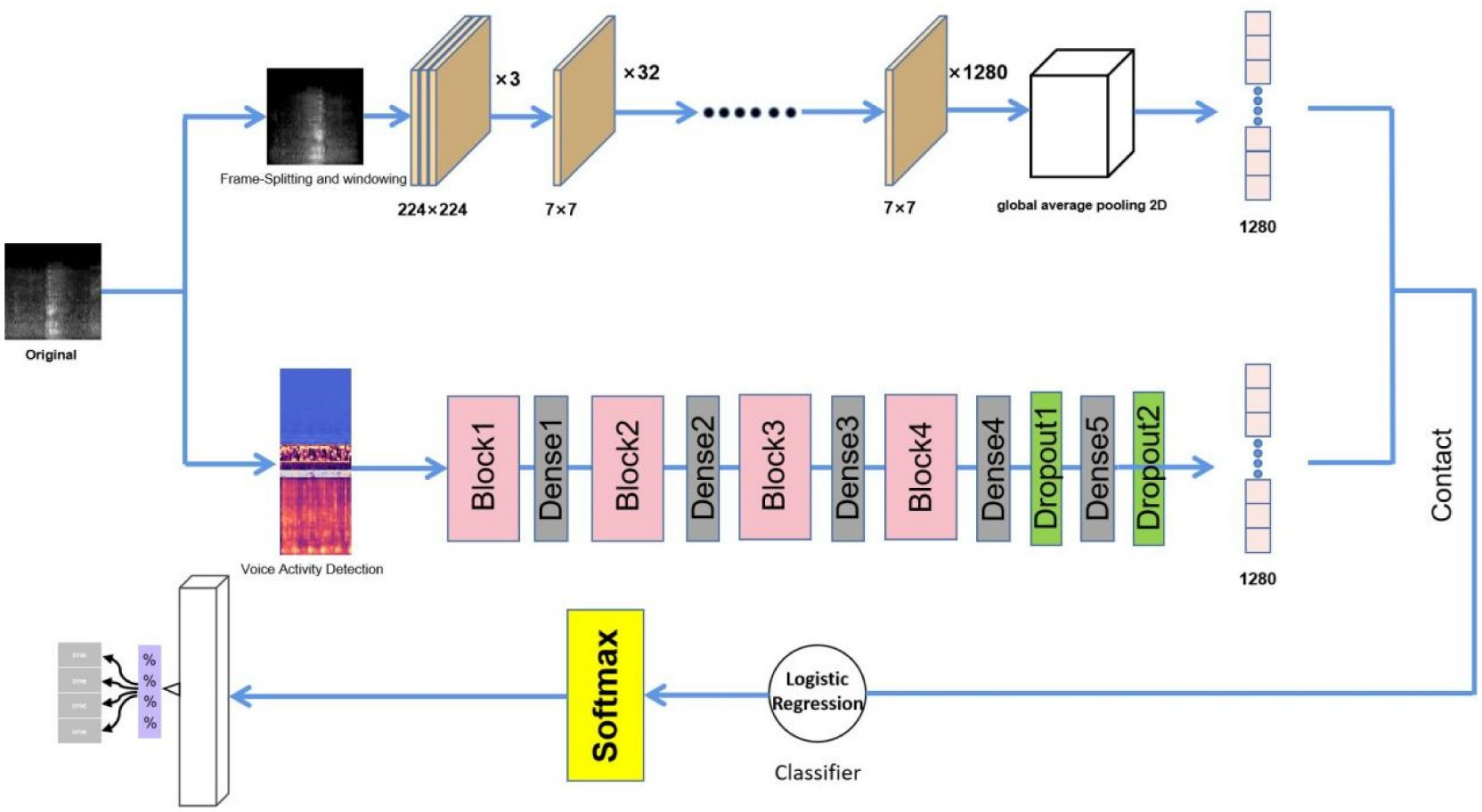
Determine the network structure and inputs and outputs of the upper and lower parallel models.

The ROC (Receiver Operating Characteristic Curve) curve is used to evaluate the classifier of each model. From Fig 11, we can observe that the area under the curve of EfficientnetB1+GRU+SVM is the highest, which means that in this case a positive sample as well as a negative sample is randomly selected and the classifier The higher the probability that the value of the positive sample is higher than the negative sample, the higher the accuracy of the classifier, and the final network structure is shown in Fig 12. In the lower layer network, a Block represents two recurrent network layers.

**Fig 13:**
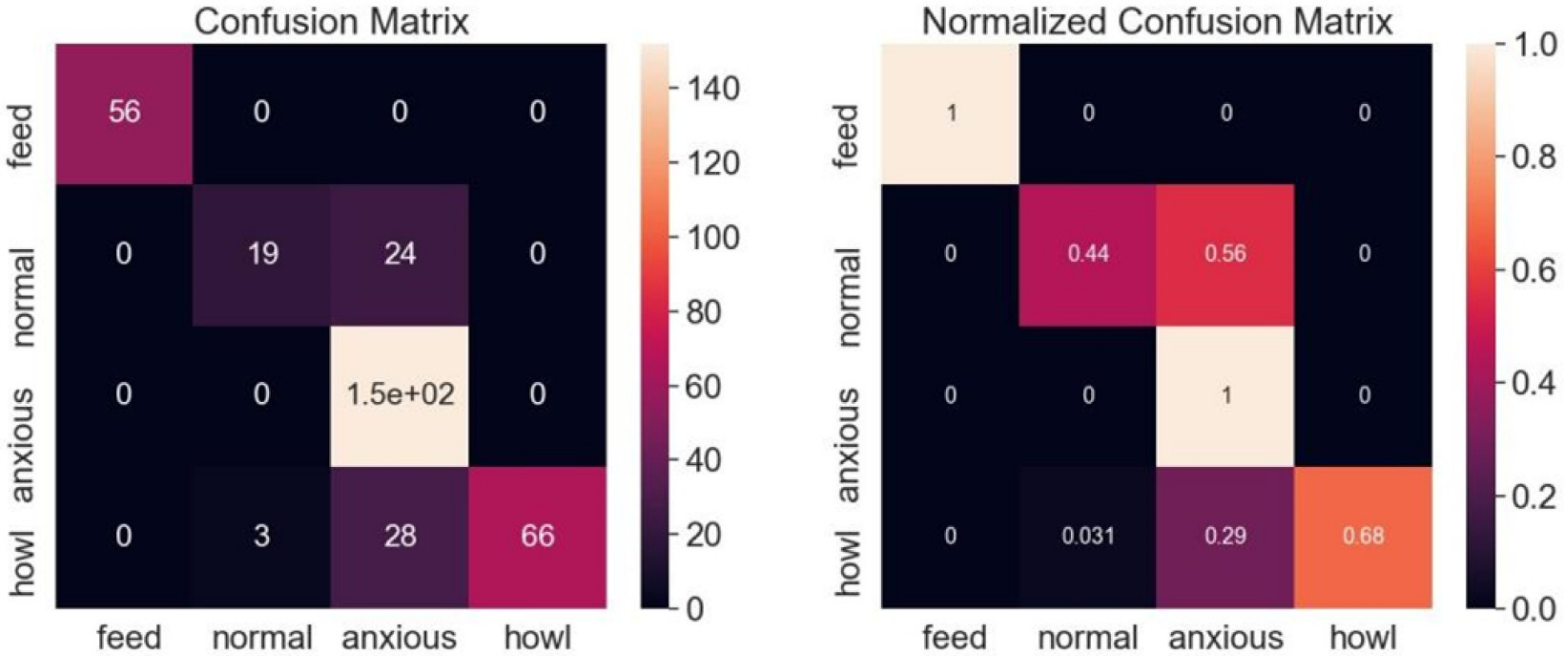
Confusion matrix diagram.

To see the effectiveness of the algorithm more intuitively, we visualized the confusion matrix for the test data. While verifying the effectiveness of the algorithm, we limit the number of test data to make the display more intuitive. During testing, we used a model with a final accuracy of 94.52% for testing and generated visualization results by extracting the data. The results of the test confusion matrix are shown in Fig 13.

The confusion matrix visualizes the errors that occur in the prediction process, as the correct predictions all appear on the diagonal. As you can see from the graph, the test results of feed and howl data performed perfectly, while the normal data had 3 misclassifications. Because the anxious class data behaves more sharply and is more transient in its acoustic characteristics, resulting in the worst test results for the anxious class data. Overall, the method proposed in this paper is effective in accurately predicting all four types of data, rather than just performing well on one type of data, as we would expect. It is clearly impractical to test models to accurately predict every data, and such overfitting tends to show worse results in practical other types of data applications. The results of this experiment also remind us that how to accurately identify sharp and short sound tasks is also an area where we can over make improvements.

### Comparison experiments

To verify the effectiveness of the proposed method in this paper, we selected the speech data of beluga whales, as well as cats and birds, from other publicly available animal sound databases (URL:https://www.xeno-canto.org/). It is worth noting that the vocal features of animals are different due to different animal species, resulting in different sound data for different animals. The average duration of the voice data provided by the site was about 2–9 s. To ensure the effective use of the voice data features of different species of animals, we padded the data length to 10 s, retaining all its feature data. The comparison results obtained by putting the data using the methods mentioned in this paper are shown in Table 5. The numbers in parentheses represent the classification of the selected animal sound recognition task.

**Table 5:** On the performance of fusion models tested on different animal sound datasets.

Data Category	Acc	Auc	Recall	Precision	F1
pig(4)	0.9339	0.9837	0.9052	0.9080	0.9047
cat(3)	0.8527	0.8927	0.8523	0.8220	0.8320
bird(8)	0.9550	0.9376	0.8692	0.8995	0.8704
bird(16)	0.9212	0.8716	0.8389	0.8528	0.8586
whale(4)	0.9064	0.8720	0.8512	0.8689	0.8494

As shown in Table 5, the fusion model proposed in this study still has excellent performance in classification recognition on other animal speech datasets, especially in the bird speech classification task with recognition accuracy of 95.50% 92.12% for the two types of data, respectively. The effect was slightly worse in Moby Dick’s voice recognition task, but it was still 90.64% and had little impact on the data. The performance in the cat data set was poor, at 85.27%. Considering from the data level, the speech data of different categories of birds and pigs are distinctly different and have longer wavelengths, and the effective feature distributions are easily captured by the proposed method in this paper. However, the sound variation of beluga whales and cats was less repetitive and less differentiated leading to a slightly poorer modeling effect. Through different experiments, it is proved that the research method can effectively extract animal sound signals with different feature signals, and the accuracy of animal speech classification task is significantly improved, with strong compatibility to different data, which reflects the robustness and robustness of this research model. However, there is still room for improvement in voice recognition of different species of animals.

## Summary of the discussion

Accurately grasp the growth status of pigs to ensure the health of pigs is a hot issue in modern intelligent agriculture, based on this, sound as one of the important physical information of pigs, using the information contained in the sound of pigs to determine the current status of pigs is of great significance to solve such problems.

This paper firstly introduces the current research status and application fields of speech recognition at home and abroad, and shows the importance of research on speech recognition algorithms for pigs in the context of the current situation and needs of animal speech recognition. In this paper, two mainstream methods for solving speech recognition tasks, spectrogram-based classification and speech feature-based recognition, are used simultaneously for speech data, and are combined on the basis of both algorithms by training a new classifier. After experiments, it is demonstrated that the fused algorithms have better results compared to individual algorithms, and the advantages and disadvantages of eight different speech features are also explored, and the capabilities of three sequence networks, RNN, LSTM, and GRU, in solving this task are analyzed. The main highlights are as follows:

1. In this paper, a fusion network idea is proposed to better solve the speech recognition task of pigs through the mutual complementation of spectral features and audio features.
2. Data preprocessing methods and comparison of advantages and disadvantages regarding endpoint detection and framing with windows are presented in the spectrogram classification section.
3. In the spectral features section, chroma, spectral contrast, tonnetz, MFCC, and LM features are extracted and combined to obtain MC, LC, and MLMC features, a total of eight different features to ensure the diversity of features and the robustness of the model.
4. A dataset of pig speech was produced, which provided raw material for other research scholars.

In addition, a large number of experiments have been conducted in this paper:

1. Seven image recognition models such as Desnet,Resnet50,Resnet152,Effcienet B1,Effcienet B2,VGG16,VGG19 were trained to ensure the best results for the selected spectrogram classification network part. Two preprocessing methods, endpoint detection and frame splitting with windowing, are also judged.
2. In the feature selection section, the eight types of selected features are experimentally compared to select the feature that contains the richest sound information.
3. Three types of sequence networks, RNN, LSTM, and GRU, are experimented separately to ensure the effectiveness of the audio recognition network part.
4. Experiment on the constructed fusion network and determine the optimal network model through the selection of different classifiers.
5. Four sets of comparative experiments were done with the existing public data set of animal speech to demonstrate the feasibility and superiority of the proposed method in this study.

The method proposed in this paper performs well in solving the pig speech classification task. Although the fusion network shows strong advantages in the feature extraction and fusion of audio data, the running time will inevitably increase due to the simultaneous operation of two different networks. In addition, a relatively large amount of computation is required in the process of running on the network, which is also one of the limitations of this research method. We are also carrying out further work around this problem to realize that the fusion network can also perform well on lightweight devices. The goal of completing the task, I believe we will completely solve this problem in the near future.

In summary, this paper demonstrates the high accuracy of the proposed fusion network. However, the algorithm proposed in this paper still has some shortcomings, firstly, the dataset is small in variety, and at the same time, since the dataset is all collected and produced by our research team, there may be individual point labeling errors. In this paper, the experimental subjects are single pigs, and there are few cases of sound overlapping together, so this paper does not consider the case of multiple pigs vocalizing at the same time, but in real life, pigs are kept in captivity. Different animals’ sound cannot be applied to different species due to different features of the trained model, and it is an important improvement direction to study the model with stronger generalization ability. This is also one of the most important development directions in the future, and we will continue to conduct related research to focus on such problems.
